# Environment-Aware Adaptive Reinforcement Learning-Based Routing for Vehicular Ad Hoc Networks

**DOI:** 10.3390/s24010040

**Published:** 2023-12-20

**Authors:** Yi Jiang, Jinlin Zhu, Kexin Yang

**Affiliations:** Department of Communications Engineering, Harbin University of Science and Technology, Harbin 150080, China; zjl2661805580@163.com (J.Z.); yangkx0101@163.com (K.Y.)

**Keywords:** VANET, multiple metrics, reinforcement learning, environment-aware

## Abstract

With the rapid development of the intelligent transportation system (ITS), routing in vehicular ad hoc networks (VANETs) has become a popular research topic. The high mobility of vehicles in urban streets poses serious challenges to routing protocols and has a significant impact on network performance. Existing topology-based routing is not suitable for highly dynamic VANETs, thereby making location-based routing protocols the preferred choice due to their scalability. However, the working environment of VANETs is complex and interference-prone. In wireless-network communication, the channel contention introduced by the high density of vehicles, coupled with urban structures, significantly increases the difficulty of designing high-quality communication protocols. In this context, compared to topology-based routing protocols, location-based geographic routing is widely employed in VANETs due to its avoidance of the route construction and maintenance phases. Considering the characteristics of VANETs, this paper proposes a novel environment-aware adaptive reinforcement routing (EARR) protocol aimed at establishing reliable connections between source and destination nodes. The protocol adopts periodic beacons to perceive and explore the surrounding environment, thereby constructing a local topology. By applying reinforcement learning to the vehicle network’s route selection, it adaptively adjusts the Q table through the perception of multiple metrics from beacons, including vehicle speed, available bandwidth, signal-reception strength, etc., thereby assisting the selection of relay vehicles and alleviating the challenges posed by the high dynamics, shadow fading, and limited bandwidth in VANETs. The combination of reinforcement learning and beacons accelerates the establishment of end-to-end routes, thereby guiding each vehicle to choose the optimal next hop and forming suboptimal routes throughout the entire communication process. The adaptive adjustment feature of the protocol enables it to address sudden link interruptions, thereby enhancing communication reliability. In experiments, the EARR protocol demonstrates significant improvements across various performance metrics compared to existing routing protocols. Throughout the simulation process, the EARR protocol maintains a consistently high packet-delivery rate and throughput compared to other protocols, as well as demonstrates stable performance across various scenarios. Finally, the proposed protocol demonstrates relatively consistent standardized latency and low overhead in all experiments.

## 1. Introduction

With the increasing demand for intelligent transportation systems (ITSs) to enhance road-traffic efficiency and reduce traffic accidents, vehicular ad hoc networks (VANETs) are expected to attract more interest in research and industry. They leverage communication between vehicles for information exchange and collaborative operations, thereby offering significant benefits in areas such as traffic management, sharing road-condition information, traffic-flow optimization, and emergency communications [1,2,3,4,5]. Over the past decade, many studies have attempted to optimize the performance of VANET routing protocols [6,7,8,9]. Despite the utility of VANETs, they come with limitations and challenges. In VANETs, data transmission faces various difficulties, including frequent changes in network topology, intermittent link connectivity, abundant environmental interference, limited channel resources, and more. These challenges can significantly impact the user experience of vehicular applications and may not meet diverse communication service-quality requirements.

While there are many traditional mobile ad hoc network (MANET) routing protocols to choose from, the challenges posed by VANETs are often insurmountable for traditional routing approaches. On complex urban roadways, vehicles typically move freely at speeds of approximately 50 km/h. The transmission paths in VANETs require support for multihop communication [10], and, as a result, many traditional routing protocols need to maintain paths from source to destination. When vehicles move rapidly, the frequent changes in network topology further increase the maintenance cost of the entire network, thereby exacerbating network congestion and making it difficult to establish effective communication links.

The applications of VANETs demand reliable and robust data transmission. Routing protocols have a crucial impact on the network’s stability and performance. Location-based routing protocols [11,12] hold significant development potential in dynamic network environments. By utilizing the global positioning system (GPS) or digital map to acquire their location coordinates and enable the sharing of location information among nodes, routing based on location can mitigate the impact of high node mobility. It possesses simplicity and scalability features. However, in urban networks, location-based routing protocols also suffer from frequent link failures and routing voids, thereby limiting the achievement of better performance.

In routing, forwarding nodes need to compromise multiple factors to select an appropriate next-hop relay towards the destination node. Choosing the right relay nodes can reduce the number of hops, transmission delay, and packet loss from the source node to the destination. Traditional location-based routing employs a greedy gorwarding (GF) strategy [13], which satisfies the requirements for low hop count and low latency. However, the high-speed movement of vehicles leads to nonuniform node distribution and communication-link disruptions. Additionally, the urban road structure imposes constraints on the topology of vehicular networks. The presence of intersections, as well as shadow attenuation caused by vegetation and buildings, significantly reduces signal strength, thereby impacting routing performance. Therefore, location-based routing protocols face challenges in adapting to the highly dynamic topology and complex working environment of VANETs.

**Figure 1:**
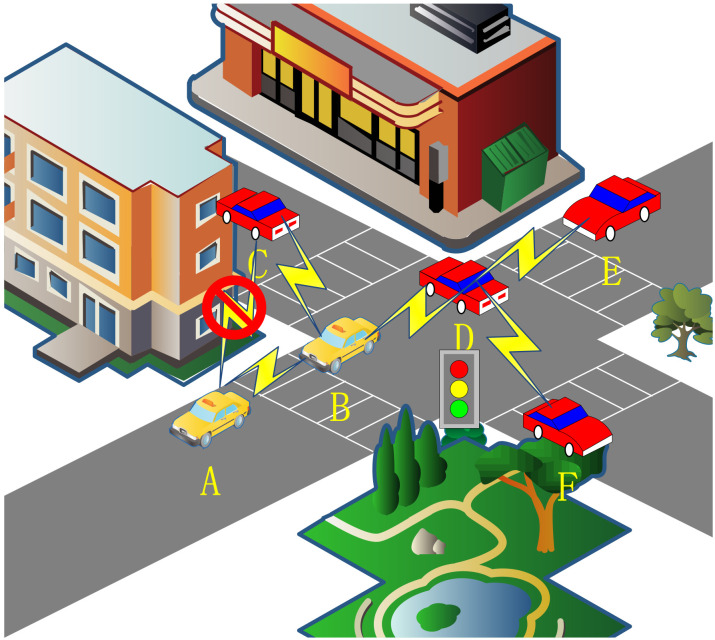
An example of part of the problem with VANETs.

Figure 1 shows the routing challenges present in VANETs. Therein, vehicle A and vehicle B are located in the effective communication range with each other, but the building restricts the possibility of nodes communicating with each other and needs to be relayed through vehicle B. Vehicle E waits to pass due to the presence of traffic lights and is disconnected from vehicle B in the routing table. However, the routing table may not be updated in time, thereby resulting in packet loss. Vehicle F travels in the opposite direction of vehicle D, thereby resulting in short link life and unstable communication. Therefore, an intermediate vehicle should be selected to forward the data.

For the limitations of traditional routing, many studies have proposed traffic-aware routing protocols [14,15,16] aimed at enhancing adaptability in urban routing scenarios. In urban traffic environments, vehicular networks are influenced by road structures and the distribution of buildings. These protocols evaluate traffic flows based on road segments and select the next relay at intersection areas. Traffic-aware routing can adapt to various network and traffic conditions, thereby offering higher efficiency. It can avoid forwarding packets to roads with low vehicle density or poor communication-link quality. Vehicles at intersections send collector packets (CPs) to gather network and traffic conditions on different road segments, which is a widely used approach in current traffic-aware routing. However, the continuous measurement process introduces additional communication overhead, thereby increasing expenses and delays. Moreover, the accuracy and update frequency of link assessments significantly impact the performance of traffic-aware routing protocols.

The development of artificial intelligence is driving advancements across various research domains, with machine learning, deep learning, and reinforcement learning serving as crucial areas within the artificial intelligence field. These research domains have had far-reaching impacts across various sectors. Notably, significant achievements have been realized in fields such as transportation systems, autonomous driving, medical-image analysis, and natural-language processing [17]. To address the challenges of dynamic routing, an increasing number of studies have turned to the use of reinforcement learning (RL), because it can autonomously learn the dynamic characteristics of networks to improve system performance while optimizing available network resources. However, vehicle behavior is characterized by a large number of states and high uncertainty, thereby making it challenging to train network models for accurate predictions. As a result, algorithms have become increasingly complex and difficult to converge.

Q-learning [18] stands out as a classic algorithm in the realm of reinforcement learning and has found widespread application in packet routing. The Q-learning algorithm has been effectively employed in the design of various types of ad hoc networks, thus encompassing VANETs, among others [19,20,21,22]. It possesses the capability to make optimal decisions through continuous interaction with the environment, even in the absence of prior knowledge about the environment. In the routing process, to ensure the selection of the correct relay nodes, a Q-Learning-based routing protocol needs to incorporate certain constraints in its design, such as speed, delay, network load, link lifespan, and others. In VANETs, the entire network can be modeled as an environment, and vehicles or data packets can be regarded as agents based on design requirements. Whether it is the forwarding of control packets or data packets, it can be seen as an interaction between agents and the environment. Through this interaction, the protocol learns the network’s latest state and continuously adjusts its forwarding strategy to ensure the reliability of link communication.

This study introduces a novel environment-aware reinforcement learning-based routing (EARR) protocol. It effectively addresses the high mobility of vehicular nodes and environmental variability. In the EARR protocol, we not only depart from the fixed-parameter form of traditional Q-learning, but also abandon the convergence method of using the optimal trajectory. The proposed protocol adjusts its behavior based on environmental feedback, thus taking into consideration metrics such as the position, speed, bandwidth, and power of the next-hop relay nodes. Most Q-learning-based approaches employ fixed learning rates and discount factors, which may prove inadequate in unstable working environments. Consequently, the accuracy of their routing selection decreases, and the links established with selected neighboring nodes become exceedingly fragile. Therefore, the EARR protocol adaptively adjusts the learning rate and discount factor based on relevant metrics.

In a dynamic network environment, the faster the network topology is changing, the more sensitive it is to environmental variations, and therefore, the learning rate should be larger. For the discount factor, which reflects future expectations, it should be smaller in more unstable environments. Additionally, traditional Q-learning methods require a significant amount of time to explore and exploit the environment when searching for the optimal trajectory, thereby leading to the exploration–exploitation dilemma. The exploration–exploitation dilemma in reinforcement learning refers to the challenge of finding the right balance between exploring new possibilities and exploiting known information to maximize cumulative rewards. Therefore, this study leverages a beacon mechanism and the acquired neighbor information to rapidly explore potential optimal or suboptimal paths. The main contributions of the proposed environment-aware reinforcement learning-based routing Protocol in this paper are as follows:

- Q tables instead of routing tables: This study proposes an environment-aware reinforcement learning-based routing (EARR) protocol for VANETs. EARR employs Q-learning for multimetric routing optimization and does not rely on fixed routing vector tables. Instead, it utilizes a continuously adaptive updated Q-value table. As a result, there is no need to maintain end-to-end links, thereby reducing the significant network overhead caused by routing maintenance. Given the highly dynamic topology of VANETs, ensuring route stability in a timely manner often leads to prohibitively high maintenance costs. By selecting the next-hop relay node based on the Q value, only local one-hop links need to be maintained, thereby forming optimal or suboptimal routes in the process of selecting the best next-hop for each hop.
- Adaptive Q-learning: In highly dynamic VANETs, link stability is extremely fragile. The Q-learning approach rapidly comprehends the network environment through beacons and utilizes neighbor information alongside adaptive Q-learning techniques to autonomously adjust the learning rate and discount factor. By altering the learning rate and discount factor, the routing adapts itself to the current environmental changes.
- Broadcast updates: In this study, EARR updates Q-table values by using a special broadcast beacon. The feasibility of the scheme is proved by building a simple vehicle Q-value update model. It also explains the features of the routing scheme, such as solving the dilemma of Q-learning development and exploration and adjusting the parameters in time under the change in environment.
- Shadow fading and residual bandwidth: In the unique working environment of VANETs, factors such as urban vegetation and buildings significantly affect signal propagation, thereby resulting in substantial signal loss and reduced link lifetimes. To mitigate data packet loss caused by low-quality communication links, this study introduces the received signal-strength indication (RSSI) into the discount factor. Due to the nature of wireless communication, signals need to be transmitted in specific frequency bands. The network is susceptible to congestion due to excessive traffic, thereby hindering network performance improvement. Therefore, we incorporate residual bandwidth estimation during the link establishment process to assess network conditions and balance network load.

The rest of this paper is ordered in the following way. Section 2 reviews the existing research work on routing protocols in VANETs. Section 3 provides the problem statement, motivational scenarios, network models, and an analysis of VANET’s Q-learning framework. Section 4 describes our routing protocol in detail for each aspect. In Section 5, the performance of EARR is evaluated through simulations and subsequently compared with other routing protocols. Finally, Section 6 concludes this paper.

## 2. Related Work

With the development of intelligent transportation systems (ITSs), the significance of vehicular networks has gained prominence. Ensuring accurate data transmission is of paramount importance, and undoubtedly, routing protocols play a pivotal role in this regard. In numerous past research endeavors, many scholars have proposed various solutions to address routing challenges within VANETs. Subsequently, this discussion will encompass a tripartite review of routing protocols encompassing the following: traditional mobile routing, traffic-aware routing, and machine learning-based routing.

### 2.1. Traditional Mobile Routing

Classic mobile routing protocols in vehicular networks include the ad hoc on-demand distance vector (AODV) [23,24], dynamic source routing (DSR) [25], the destination-sequenced distance vector (DSDV) [26], and greedy perimeter stateless routing (GPSR) [13]. The DSDV routing protocol is a proactive routing protocol where every node in the network needs to maintain up-to-date routing information. This increases network overhead and requires frequent updates of the routing information due to the constantly changing topology. The AODV and DSR are on-demand routing protocols, and their simulation results in depicting various aspects that have not been satisfactory in rapidly changing network environments. Additionally, due to the internal maintenance mechanisms of these routing protocols, they can cause network congestion, consume significant bandwidth, and even degrade network performance [27,28].

Compared to previous routing protocols, location-based geographic routing is considered to be one of the best solutions for dynamic network topologies. In protocols employing a greedy strategy, vehicles forward data packets to the next hop that is closest to the destination node based on their neighbor table. GPSR is a classic location-based routing protocol that combines greedy forwarding with perimeter forwarding. Vehicles determine their own positions and broadcast their state information among neighboring nodes. GPSR offers low latency and control overhead characteristics. However, GPSR does not consider parameters such as beacon intervals, node velocities, or movement directions, which can lead to link instability and limited routing performance.

### 2.2. Traffic-Aware Routing

The traffic-aware routing protocol can adapt to different network and traffic states. In VANETs, vehicles serve as the entities responsible for forwarding data packets from source to destination. As a result, traffic-aware routing protocols make routing decisions based on the availability of road segments and the reliability of intervehicle links. This ensures the efficiency and quality of data-packet transmission. Traffic awareness improves the intelligence of route selection, thereby catering to different road conditions and traffic states and making communication within VANETs more reliable and efficient.

In [29,30], the authors enhanced the improved greedy-forwarding mechanism by selecting neighbors with high received signal-strength indicator (RSSI) values. However, in areas near intersections, vehicles often change direction rapidly, which can lead to the selection of the wrong next hop. Furthermore, neighbor location predictions may be inaccurate, as the instantaneous movement information received by the beacons may not accurately depict the motion state of the vehicles over a period of time. This can lead to unreliable next-hop predictions.

GyTAR [31] is a geographic routing protocol based on intersections, which is designed to find optimal routes in urban environments. GyTAR dynamically and sequentially selects intersections through which data packets are forwarded towards the destination. When packets were transmitted to an intersection, the routing decisions are made based on parameters such as changes in traffic flow in the surrounding road segments and the remaining distance to the destination from the next intersection. In GyTAR, within segments between intersections, data forwarding is achieved using an enhanced greedy-forwarding mechanism.

In [32], the proposed LITAR is a lightweight traffic-aware routing protocol that relies on intersections to determine the transmission direction for packets. To reduce the additional network overhead generated during segment connectivity measurements and maintain the accuracy of measurement time, LITAR introduces two new algorithms: enhanced validity period calculation (EVPC) and restricted collector packet reply (RCPR). Additionally, LITAR makes routing decisions based on factors such as vehicle direction density, road-network connectivity (RNC), and distance to the destination. In [33], a reliable traffic-aware routing protocol was introduced, which leverages the real-time traffic and network-state measurement (RTNSM) process introduced in [32] to achieve accurate and lightweight road-state assessment. This protocol selects the next hop based on the road structure, predicted positions of neighbors, received signal strength, and recency of mobility information received from neighbors.

TLRP [34] is a traffic-aware and link-quality-sensitive city vehicular network routing protocol. The protocol introduces a novel routing metric, namely link transmission quality (LTQ). Considering the high mobility of vehicles, nonuniform distribution, and the quantity and quality of the communication links along the routing path, the protocol prevents packet forwarding in situations of severe congestion in ultra-low density road sections or vehicle networks. Subsequently, a road weight evaluation scheme was proposed that utilized LTQ and link information to assess each road segment. IQRRL [35] is a quality-of-service (QoS) routing protocol based on intersections. IQRRL routing involves the selection of intersections within the chosen road and the choice of the next hop. IQRRL takes into account fundamental metrics such as latency and connection probability. It utilizes the Dijkstra algorithm to consider the communication quality from adjacent roads to the destination node, thereby avoiding the problem where local optimization leads to intersections getting farther away from the endpoint. Finally, reinforcement learning is employed to establish a network among vehicle nodes within the road, thereby significantly enhancing the stability and reliability of message transmission.

### 2.3. Machine Learning-Based Routing

Machine learning is now widely applied in various research domains and has yielded numerous achievements. The authors of [36] employed the support vector machine (SVM) to optimize node selection, thereby utilizing the SVM to process vehicular data and categorize nodes into excellent next hops and ordinary next hops. And they generated routing metrics to determine routing indicators and their corresponding weight factors. In [37], a context-aware reliable routing protocol was proposed, which combines k-means clustering and the support vector machine (SVM). The k-means clustering divides routes into two categories: those with a high mean square error (MSE) are labeled as BAD, while those with a low MSE are labeled as GOOD. After training on routing data, the SVM enhances the performance of various metrics and routing efficiency. The SVM is a supervised machine-learning method that requires the training of data. However, because vehicle states are constantly changing, the training dataset needs to be large, thereby making the algorithm relatively complex.

Reinforcement learning is a branch of machine learning that infers the external environment through a series of decision processes. In routing algorithms that introduce reinforcement learning, each data packet acts as an agent, and the network serves as a learning environment. The state set involves all network nodes, and the action set consists of all single-hop neighbors that the agent can choose in the current state. In PFQ-AODV [38], a link evaluation technique based on fuzzy logic was designed to analyze the link status between vehicles and neighboring vehicles. This link assessment was used to update the Q value in PFQ-AODV. The neighbor node with the highest Q-value is selected as the best next hop. As the number of vehicles increases, updating and maintaining the Q table requires significant communication overhead and computational resources. In [39], a VANET routing approach based on Q-learning and Qgrid was proposed. This method operates at both the macro and micro levels. At the macro level, the network is divided into smaller grids. The Q-learning algorithm is then used to learn traffic-flow characteristics within these grids, and Qgrid explores the most suitable next grid in the target direction. It utilizes policies provided at the micro level to select relay vehicles within each grid.

QTAR [16] is a novel traffic-aware routing protocol based on RSU-assisted Q-learning. It employs two Q-learning-based routing algorithms for sending data packets between vehicles at the road and between RSUs at the intersection. During the routing process, vehicles broadcast HelloV2V messages, including their speed and position. Additionally, RSUs exchange HelloR2R messages with each other. Each vehicle maintains a V2V Q table for sending data packets on road segments. This Q table is refreshed upon receiving any HelloV2V message. In the routing algorithm based on R2R Q-learning, each data packet serves as a learning agent, and the state set represents neighboring RSUs. Furthermore, the action space encompasses all the adjacent intersections. Each RSU maintains two Q tables: a V2V Q table for sending data packets on road segments and an R2R Q table for sending data packets at intersections.

In [40], a context-aware edge-based vehicular network packet forwarding scheme was proposed. It utilizes a fuzzy logic-based edge-node selection protocol to search for optimal edge nodes in a distributed manner, thereby facilitating the efficient utilization of wireless resources for data-packet forwarding through the edge. Reinforcement learning algorithms are employed to optimize communication within the last two hops, thereby enhancing the adaptive capabilities of communication routing. Ref. [20] presents a geographic routing solution within a layered routing framework. It primarily consists of two components: a crossroads-optimal road-segment-selection routing strategy based on multidimensional Q-learning and a greedy routing strategy for selecting the best relay node within the chosen road segment. In IV2XQ, the state space represents all the network intersections, which effectively reduces the number of states for Q-learning, thereby making it more feasible. However, in this approach, a central server explores the network environment based on historical traffic data and may not adapt well to highly stochastic real-world environments. In [41], the IRQ also employs a central server for training. To prevent network congestion, the IRQ controls traffic through traffic management. Roadside units (RSUs) monitor road conditions to understand traffic situations. This approach uses both global and local views during routing, thereby creating a traffic propagation mechanism. This mechanism helps the IRQ update traffic information and provides a fresh global view to the network server. The global view is used for designing Q-learning-based routing techniques, while the local view designs a greedy routing strategy for each road segment to discover the best next-hop nodes.

## 3. Preliminaries

This section presents the preliminary knowledge of our research, including the problem statement, motivation scenario, network model, and Q-learning framework to promote understanding of the EARR.

### 3.1. Problem Statement

The main research problems considered in this study can be summarized as follows:

- Many routing algorithms based on ML or RL are inherently complex. The intricate urban road structures and constantly changing vehicle conditions further exacerbate the challenges in program execution. These algorithms confront high levels of uncertainty, thereby making it difficult to achieve satisfactory results in the end.
- In VANET routing protocols based on Q-learning, many protocols rely on RSUs to forward data or monitor road traffic at intersections, which is difficult to meet in many practical scenarios.
- Many routing protocols cannot detect network congestion. The routing protocol should be able to select the forwarding according to the busy status of the next-hop node to improve the network performance.
- The protocol needs to adjust parameters in real-time based on the vehicle environment to prevent performance degradation due to the environment’s dynamic changes.

### 3.2. Motivation Scenario

To address the limitations of the previous research, the study introduced an adaptive perception V2V routing protocol based on Q-learning. In this protocol, we broadcast our own state through beacon packets, and receiving nodes extract information about neighboring nodes, including vehicle IDs, positions, received signal strengths, available bandwidth, and other relevant parameters.

By extracting beacon data from neighboring vehicles, training the Q table, and selecting vehicles with the maximum Q values, this study identifies the optimal or suboptimal paths. This routing approach eliminates the need for additional RSUs or infrastructure, thereby reducing the hardware requirements in the working environment. Compared to RL routing protocols that partially rely on the geographical environment as states [16,20,39,41], the entire algorithm is relatively easy to implement. For example, the protocol needs to divide the network environment into grids and choose the next hop based on forwarding policies within the optimal next grid. However, grid division heavily relies on the urban environment, as intersections and buildings within the grids affect signal transmission. Alternatively, it depends on forwarding data at intersections, assessing road conditions, and then forwarding packets to the evaluated optimal road segment. This method mitigates sudden changes in vehicle direction and signal attenuation caused by building obstruction. However, its reliance on RSUs and traffic-aware messages restricts its applicability.

**Figure 2:**
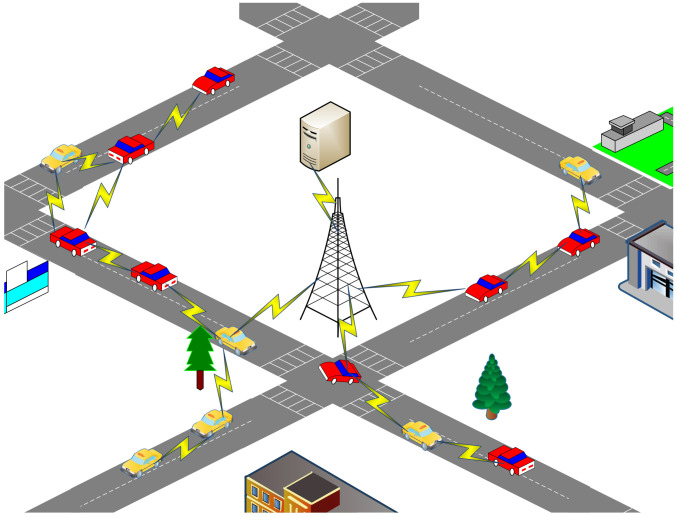
VANET scenario.

This study describes a VANET scenario that comprises a vehicle network and a base station (BS) for real-time traffic information, road conditions, and accident reports, as depicted in Figure 2. The BS serves as the destination node, while the vehicles distributed across various road segments are considered as the source nodes for data transmission. Data packets are relayed through other vehicle nodes. Vehicles report their own driving status, traffic conditions on their respective road segments, and any sudden accidents they encounter.

The BS is responsible for collecting vehicle and road-condition data and uploading it to a traffic management server, which facilitates various research or application platforms such as traffic-flow optimization, emergency assistance, and route planning.

This application scenario considering the design of a distributed data collection server that aligns with geographical distribution characteristics. For instance, a city map can be divided into multiple regions, with each region assigned to an RSU or BS for data collection. Each server would be connected to RSUs or BSs and be responsible for one or more regions. The division of regions can follow a cellular structure design; further design details are beyond the scope of this paper’s discussion.

The working environment of VANETs inherently exhibits high levels of randomness and complexity. Designing routes that align with the current application scenarios is extremely challenging, and these challenges require special consideration when devising effective communication and data-transmission solutions. For instance, the high dynamism of vehicles necessitates routing algorithms to rapidly adapt to changes in the network topology. Routing algorithms must effectively address unreliable communication environments, including issues such as signal obstruction, multipath propagation, and signal attenuation. Sensitive information is involved in self-organizing networks; hence, routing algorithms must account for privacy protection and security concerns. Effective spectrum resource management is crucial to support communication among a large number of vehicles. Additionally, meeting the unique requirements of vehicular networks may necessitate crosslayer design and other related challenges. This research places a particular emphasis on the dynamism of vehicles, shadow fading, and effective bandwidth to identify the most suitable paths between source and destination nodes. The proposed protocol is envisioned as a means to relay data in distributed information applications and services that require multihop communication within vehicular networks. The goal is to enhance data delivery rates and reduce network congestion. The design model we have proposed leverages the mobility of vehicles to collect data along road segments, thereby minimizing the costs associated with complex infrastructure. It simultaneously ensures the real-time and accurate diffusion of traffic information.

### 3.3. Network Model

In the network model, an adaptive framework was employed to implement packet routing in VANET scenarios. The BSs connected to upper-level servers are deployed at the central locations within the urban area. V2V packet forwarding is utilized in the intersection areas. Vehicles are equipped with OBUs for data forwarding and assumed to have GPS systems or digital maps that provide real-time geographic location information for each vehicle. The abbreviations and notations used in this paper are listed in Table 1.

The vehicle network can be considered as a directed graph G = (V, L), where V is a set of vehicle nodes, and L is a set of directed links. A link between vehicle node pairs (*v*, *w*) indicates that *v* is a sender, *w* is a receiver, and vehicle node *w* is in the transmission range of vehicle node *v*. Here, link *l* = (*v*, *w*), with *l*∈L. For a source *S* and a destination *D*, a route from the *S* to the *D* is denoted as

(1) $$\mathrm{RP}\left(src,dest\right)=\left\{src=v_{n_{1}}\to v_{n_{2}}\to \cdots \to v_{n_{m}}=dest\right\}.$$

Herein, $(v_{n_{i}}$, $v_{n_{i+1}})$∈L, ∀i = 1, ⋯, m−1. For convenience of presentation, sometimes we use the notation RP instead of RP(src,dest). The number of hops of the route RP is denoted as #hops(RP) = m−1.

When vehicles receive beacons, the Q values are adjusted based on the Q-learning training methodology. This adjustment depends on factors such as relative vehicle movement, signal strength, and network bandwidth. The path sequence is determined by selecting the neighbor node with the highest Q value in the Q table.

### 3.4. Q-Learning Framework

Reinforcement learning solves optimization problems through the specific framework of the Markov decision process (MDP) [42,43]. The MDP can be described by the tuple (S, A, P, R, γ), where S denotes a finite set of state spaces; A denotes a finite set of action spaces; P is the state-transfer probability denoted as $P(s_{t+1}|s_{t},a_{t})$, i.e., the probability of transferring after completing an action $a_{t}$ in the current state $s_{t}$ to the next state $s_{t+1}$ probability; *R* is the immediate reward; and γ is the discount factor.

Q-learning is a model-free reinforcement learning method based on the Markov decision process in which an agent selects actions to interact with the environment. Over a series of decision processes, the agent controls its behavior to maximize rewards. Unlike traditional machine learning, where training data are readily available, in Q-learning, training data are collected by the agent as it continuously explores an unknown environment. The algorithm relies on trial and error to find the optimal control strategy [44]. The entire workflow of this algorithm bears a close resemblance to the process of route selection, thereby making it applicable to routing models.

When the agent explores an unknown environment through trial and error, it maintains a Q table containing optimal state-action pairs and their respective Q values. The Q-learning algorithm adjusts action selection based on rewards obtained from the environment and evaluates the chosen actions in the current state to maximize the Q value. The iterative process of updating the Q value in the Q-learning algorithm is depicted in Equation (2). Action selection relies on the action corresponding to the maximum Q value in the Q table for the current state. However, during the action selection process, there is a need to balance exploration and exploitation, typically achieved using an ε-greedy strategy. Nevertheless, the Q-learning approach is computationally expensive for exploring optimal paths, especially in scenarios with numerous states such as VANETs.

(2) $$\begin{array}{cc} Q(s_{t},a_{t}) & \leftarrow Q(s_{t},a_{t})\\ \; & +\alpha \{r_{t}+\gamma \times \max_{a^{{}^{\prime }}}Q\left(s_{t+1},a^{{}^{\prime }}\right)-Q\left(s_{t},a_{t}\right)\}. \end{array}$$

**Figure 3:**
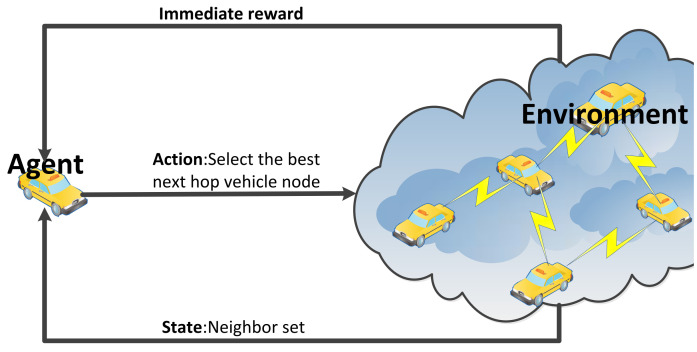
Q-learning structure for VANETs.

In the design presented in this paper, the agent corresponds to the vehicle nodes. To train the Q table and converge towards optimal paths, broadcast beacons are used to explore the network. The agent serves as the learner and decision maker in the Q-learning framework, thereby continuously interacting with the environment to learn control strategies. The environment encompasses the entire VANET scenario, or all objects that interact with the agent. It provides appropriate responses to actions and adjusts the state accordingly. Figure 3 shows the collaboration between the agent and environment in Q-learning for vehicle routing.

The state space is the set of all valid neighbor nodes of the vehicle, and $s_{t}$∈S can be depicted as $N_{v_{n_{i}}}$, thereby representing the neighbor set of vehicle $v_{n_{i}}$. In particular, the size of the state space varies for each vehicle.

The action space, the selectable next-hop neighbor nodes, and $a_{t}$∈A represent the action taken by the agent at moment *t*. In particular, since beacons are employed as actions, the broadcasted beacons are treated as reverse actions as they are received by the nodes. This is important, as it allows for fast convergence with low network consumption and a direct solution to the exploration or exploitation dilemma. The process of receiving a beacon is equivalent to exploring all possible next states in the current state.

**Figure 4:**
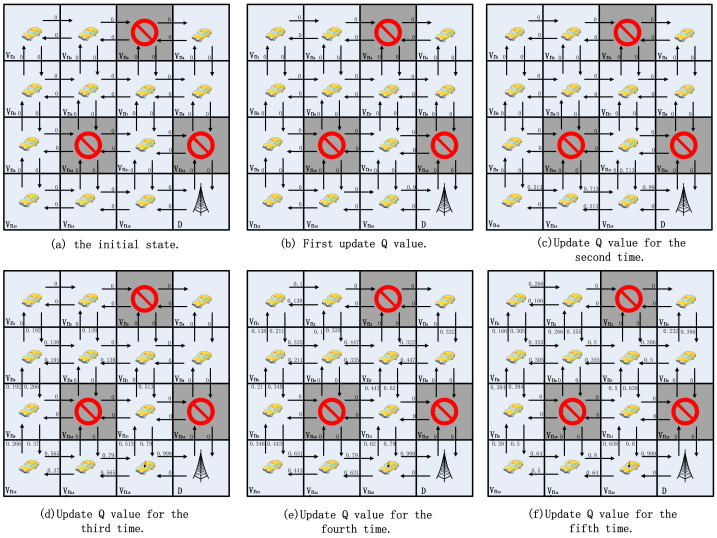
A 4 × 4 grid Q-learning update process.

As shown in Figure 4, this study gives simple examples to demonstrate how the beacon update process works to converge quickly. For simplicity, the authors assume that each grid is a vehicle, $V=\{v_{n_{1}},v_{n_{2}},\cdots ,v_{n_{15}}\}$, and that two neighboring vehicles are able to communicate directly with each other and form a link *l*, for example, *l* = ($v_{n_{1}}$, $v_{n_{2}}$). That is, there are four neighbor nodes, and the arrows indicate the selection of a neighbor agent to forward the packet. The value above each arrow indicates the Q value of the corresponding action. For simplicity, the authors assume that the vehicle has four actions, i.e., up, down, left, and right. In the current state $s_{t}$, the optimal action is defined as follows:

(3) $$a_{t}^{*}=\underset{a_{t}}{\arg\max}Q\left(s_{t},a_{t}\right).$$

In this example, if a node receives a beacon from the target node, it receives a reward of one. For beacons received from other neighboring nodes, the reward is 0. Throughout the grid model, randomly make a portion of the grid empty. That is, there are no vehicle nodes to send beacons, so the nodes around an empty node are unable to update the Q value in that direction.The Q-value update formula is as in Equation (2), with the discount factor γ = 0.8 and the learning rate α = 0.9.

In Figure 4, the lower right corner is the target grid D. Neighboring grids $v_{n_{12}}$ and $v_{n_{15}}$ can send packets to the target via one hop. That is, for the beacon sent by D, grids $v_{n_{12}}$ and $v_{n_{15}}$ are able to receive it. Grid $v_{n_{12}}$ is labeled as null here, so it is not involved in updating the Q value. From Equation (2), $Q^{\left(1\right)}\left(v_{n_{15}},v_{n_{15}}\to D\right)=0+0.9\times \left(1-0\right)$, as shown in Figure 4b. After the second global Q-value update, i.e., $v_{n_{11}}$, $v_{n_{13}}$, and $v_{n_{14}}$ Q-value update, $Q^{\left(2\right)}\left(v_{n_{11}},v_{n_{11}}\to v_{n_{15}}\right)=0+0.9\times \left(0+0.8\times \max\left\{0.99,0,0.513\right\}-0\right)$, $Q^{\left(2\right)}\left(v_{n_{13}},v_{n_{13}}\to v_{n_{14}}\right)=0+0.9\times \left(0+0.8\times \max\left\{0.713,0,\right\}-0\right)$, $Q^{\left(2\right)}\left(v_{n_{14}},v_{n_{14}}\to v_{n_{15}}\right)=0+0.9\times \left(0+0.8\times \max\left\{0.99,0,0.513\right\}-0\right)$, as shown in Figure 4c. When the third update is done, the Q values of most of the directions have been updated as shown in Figure 4d. And on the fourth time, the Q values of all the directions have been updated, and it is possible to reach the destination grid from any grid in the grid map based on the Q value, as shown in Figure 4e. The Q values will stabilize in Figure 4f. Assuming that the source is grid $v_{n_{1}}$ and taking the action with the largest Q value in its own neighbor set $N_{v_{n_{i}}}$, the route to reach the destination grid is $\mathrm{RP}\left(v_{n_{1}},D\right)=\left\{v_{n_{1}}\to v_{n_{5}}\to v_{n_{9}}\to v_{n_{13}}\to v_{n_{14}}\to v_{n_{15}}\to D\right\}$, #hops(RP)=6.

**Figure 5:**
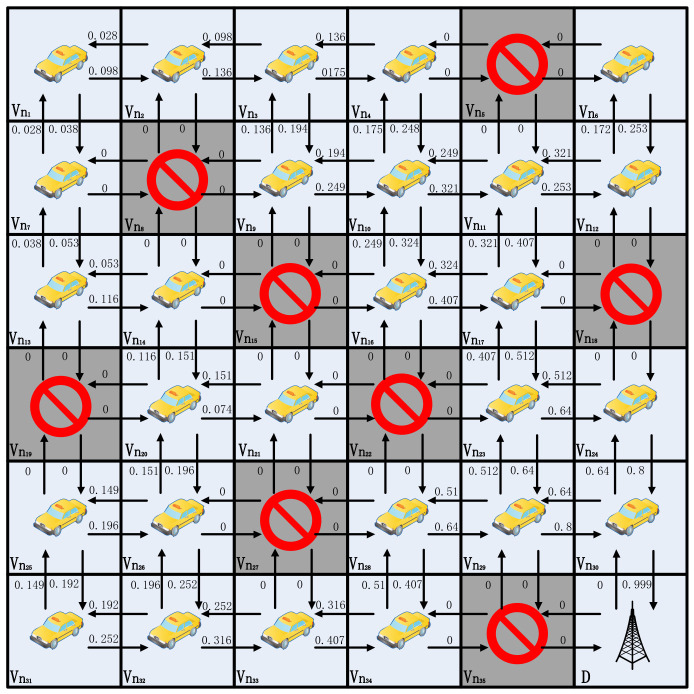
A 6 × 6 grid Q-learning update process.

Figure 4 gives a 4 × 4 model from having no a priori information on how to converge to a stable path; only four updates are needed. Figure 5 presents a 6 × 6 model, and, similarly, convergence for the 6 × 6 model typically requires six updates, thereby stabilizing around the seventh update. It is essential to note that as the model size increases, the number of updates required for convergence using this method becomes less stable. Based on the n × n model, it can be easily demonstrated that, in the most ideal scenario, completing updates in a sequential manner from the destination to the top-left corner requires only one or two updates. Conversely, in the worst-case scenario, updating sequentially from the top-left corner to the destination necessitates 2(n−1) updates. In practice, the update order is random, thereby making it unlikely for either extreme case to occur. Such convergence is undoubtedly very rapid and relies entirely on the broadcast. It is worth noting that in the learning process of the 6 × 6 model, the minimum path can be found independently. Similarly, with grid $v_{n_{1}}$ as the source node, the route to the destination grid is $\mathrm{RP}\left(v_{n_{1}},D\right)=\left\{v_{n_{1}}\to v_{n_{2}}\to v_{n_{3}}\to v_{n_{9}}\to v_{n_{10}}\to v_{n_{16}}\to v_{n_{17}}\to v_{n_{23}}\to v_{n_{29}}\to v_{n_{30}}\to D\right\}$, #hops(RP)=10 instead of $\mathrm{RP}\left(v_{n_{1}},D\right)=\left\{v_{n_{1}}\to v_{n_{7}}\to v_{n_{13}}\to v_{n_{14}}\to v_{n_{20}}\to v_{n_{26}}\to v_{n_{32}}\to v_{n_{33}}\to v_{n_{34}}\to v_{n_{28}}\to v_{n_{29}}\to v_{n_{30}}\to D\right\}$, #hops(RP)=12.

**Figure 6:**
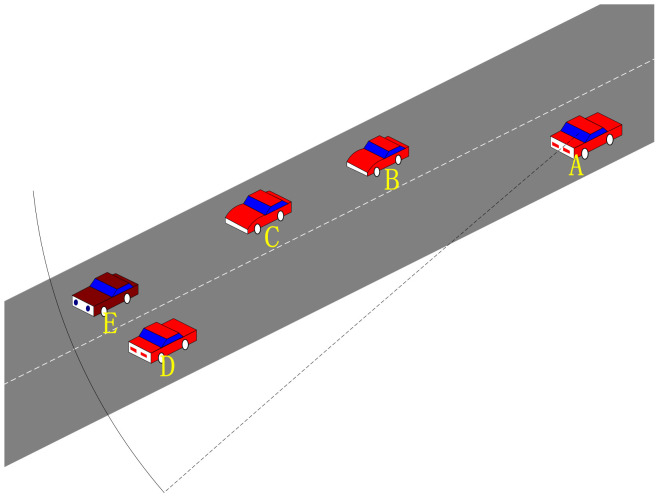
Two-lane traffic map.

Figure 6 illustrates the broadcasting characteristics in real road conditions. Vehicle nodes B, C, D, and E can receive beacons from vehicle A, and these vehicles can also transmit data to vehicle A. When vehicle D needs to transmit a data packet, its neighbor set $N_{v}$ = {A, B, C, E}, vehicle A is undoubtedly the best choice. For vehicle D, vehicle A can achieve the furthest transmission distance within its communication capability, thereby minimizing #hops(RP). The Q-learning update mechanism ensures that vehicle D selects vehicle A, because if data require a two-hop transmission, the Q value undergoes two discounts, thereby making it smaller compared to the Q value with a single discount. Therefore, vehicle A’s Q value is guaranteed to be the largest. However, for node E, vehicle A is not a good choice. Since both vehicles are moving relative to each other, they are likely to move out of communication range easily.

Unlike a simple static model, the study must deal with a highly dynamic environment. Therefore, in the process of algorithmic learning, adapting to environmental changes is of paramount importance. This adaptability enables vehicles to learn how to select appropriate routing action for different network states, thereby ensuring that data packets can be effectively guided to their destination in the ever-changing VANET environment.

## 4. Algorithm Design

This section describes the environment-aware adaptive reinforcement learning-based routing (EARR) protocol. In EARR, vehicle nodes make optimal routing decisions considering node stability, node duration, received signal strength, and available bandwidth, and the proposed adaptive scheme improves routing performance.

### 4.1. Link Stability Factor

In EARR, the link stability factor is used to adjust the learning rate α, which helps obtain more stable routes during route adjustment. The link stability factor can play an important role in the routing decision. A link l=(v,w), where l∈L. At time $t_{i}$, the positions of nodes *v* and *w* are ($x_{v}^{t_{i}}$, $y_{v}^{t_{i}}$) and ($x_{w}^{t_{i}}$, $y_{w}^{t_{i}}$), respectively. At time $t_{i+1}=t_{i}+\Delta t$, the positions of nodes *v* and *w* are ($x_{v}^{t_{i+1}}$, $y_{v}^{t_{i+1}}$) and ($x_{w}^{t_{i+1}}$, $y_{w}^{t_{i+1}}$), respectively, and the location information is collected by the beacon. $DIST^{t_{i}}$(v,w) and $DIST^{t_{i+1}}$(v,w) represent the Euclidean distance between *v* and *w* at time $t_{i}$ and $t_{i+1}$, respectively. The calculation formula is as follows:

(4) $$DIST^{t_{i}}\left(v,w\right)=\sqrt{{\left(x_{v}^{t_{i}}-x_{w}^{t_{i}}\right)}^{2}+{\left(y_{v}^{t_{i}}-y_{w}^{t_{i}}\right)}^{2}}.$$

For a link l=(v,w), we define $\left|\Delta D\left(l\right)\right|=\left|DIST^{t_{i+1}}\left(v,w\right)-DIST^{t_{i}}\left(v,w\right)\right|$ as the change in distance between node *v* and node *w* when the time is $t_{i}$ and $t_{i+1}$, respectively. The smaller |ΔD(l)| is, the more stable the distance between the two nodes *v* and *w* remains within the time interval Δt ($\Delta t=t_{i+1}-t_{i}$). Conversely, if |ΔD(l)| is larger, the distance between the two nodes *v* and *w* in the time interval Δt changes more dynamically. The link stability factor for link l=(v,w) at interval time is defined as follows:

(5) $$LSF_{\Delta t}\left(l\right)=\frac{\left|\Delta D(l)\right|}{\Delta D_{max}},$$

where $\Delta D_{max}=2\cdot sp_{max}\cdot \Delta t$, and $sp_{max}$ is the maximum node speed. Note that the more stable the distance between two nodes *v* and *w* is maintained, that is, the smaller LSF is, the longer the lifetime of the link (v,w) is maintained. Vehicles can stay in relative distance longer. The corresponding Q-learning update process does not need to be adjusted too much.

### 4.2. Link Duration Factor

Since the vehicle is moving quickly, it is important to evaluate the duration of the link l=(v,w). The link duration depends on how long the two nodes can communicate stably. For routing, a longer duration link can undoubtedly ensure stable communication services. After a node obtains the location information of itself and its neighbors, it can estimate the duration of a link.

Figure 6 shows a scenario for calculating the link duration, and *R* is the node-stable radio transmission range. ΔD(l) is the trend of the relative distance between the two nodes, and a positive value ΔD(l) represents the distance motion between the two nodes. The relative speed between the two nodes is as follows:

(6) $$RV_{\Delta t}\left(l\right)=\frac{\Delta D(l)}{\Delta t}.$$

The link duration can be calculated as follows:

(7) $$LD(l)=\left\{\begin{array}{cc} \frac{\left|R-DIST^{t_{i+1}}\left(v,w\right)\right|}{RV_{\Delta t}\left(l\right)}, & if\;\Delta D(l)>0\\ \frac{DIST^{t_{i+1}}\left(v,w\right)}{\left|RV_{\Delta t}\left(l\right)\right|}, & if\;\Delta D(l)<0\\ \frac{R}{2\cdot sp_{max}}, & if\;\Delta D(l)=0 \end{array}\right..$$

When ΔD(l)>0, the expected time for both nodes to exceed the transmission range is $\frac{\left|R-DIST^{t_{i+1}}\left(v,w\right)\right|}{RV_{\Delta t}\left(l\right)}$. When ΔD(l)<0, the time when two nodes are close to each other is calculated. The closer the two nodes are, the shorter the expected time value. Therefore, the two nodes are not suitable for data transmission. If ΔD(l)=0, LD(l) is set to a fixed value.

The link duration factor can be calculated as follows:

(8) $$LDF\left(l\right)=\left\{\begin{array}{cc} \frac{LD(l)}{n\cdot T_{B}}, & LD\left(l\right)<n\cdot T_{B}\\ 1, & \mathrm{otherwise}. \end{array}\right.,$$

where $T_{B}$ indicates the beacon interval. *n* is an adjustable value. If the LD(l) calculated by Equation (7) is less than the link-duration threshold $n\cdot T_{B}$, the linear formula is used to normalize the link duration. Proper *n* values can help the selection of forwarding nodes.

### 4.3. Received Signal-Strength Factor

Link stability depends not only on link duration, but also on signal strength. The received signal power is primarily affected by path loss and shadow fading. Path loss is caused by the dissipation of the signal and the effects of the propagation channel. Shadow fading is caused by obstacles between the transmitter and receiver, where these obstacles lead to signal power attenuation through phenomena such as absorption and reflection. When the attenuation is particularly strong, the signal becomes blocked. The impact of shadow fading is undoubtedly particularly pronounced in urban areas, where the presence of various buildings poses a significant challenge. Especially at intersections, there are significant changes in the structure of the vehicle movement, thereby leading to signal attenuation around building corners. The received signal strength comes from neighbor beacons.

The received signal strength factor can be calculated as follows:

(9) $$RSSF\left(l\right)=\left\{\begin{array}{cc} \frac{RSSI\left(v\right)-RSSI_{min}}{RSSI_{max}-RSSI_{min}}, & RSSI\left(v\right)>RSSI_{min}\;and\\ & RSSI\left(v\right)<RSSI_{max}\\ 1, & RSSI\left(v\right)>RSSI_{max}\\ 0, & RSSI\left(v\right)<RSSI_{min} \end{array}\right..$$

In Equation (9), $RSSI_{max}$ is the maximum-received signal-strength threshold, and $RSSI_{min}$ is the minimum-received signal-strength threshold. When the signal strength received by the neighbor’s beacon is greater than $RSSI_{max}$, it indicates that the working environment of the vehicle network has little interference, and a stable link can be established. Otherwise, it indicates that there is some interference between the two nodes. Here, a linear model is used to calculate the strength factor. When the value is less than $RSSI_{min}$, this object is not considered.

### 4.4. Available Bandwidth

The primary objective of many tasks is to identify viable routes from source to destination, regardless of current network traffic or application demands. Consequently, networks are susceptible to overloading due to excessive traffic, thereby resulting in applications being unable to improve their performance. To ensure access to network bandwidth, it is imperative to ascertain the available end-to-end bandwidth along the route from source to destination. End-to-end throughput is a parameter with a concave profile [45], which is determined by the bottleneck bandwidth of the intermediate hosts in the routing path. Thus, to guarantee that the data transmission has ample usable bandwidth, it is essential to consistently opt for higher bandwidth environments along the end-to-end route. However, determining the remaining bandwidth using the IEEE 802.11 MAC protocol remains a challenging issue. Here, this study adopts the method proposed by [46] for estimating the medium radio occupancy factor in the network, thereby defining the medium’s occupancy factor as the percentage of channel utilization by mobile nodes within the same interference region. By continuously monitoring the radio activity on the channel through ongoing listening, the local assessment of medium radio occupancy allows for the evaluation of available bandwidth. To obtain the medium’s occupancy rate, each mobile node maintains its occupied period within an equal time interval ($T_{BW}$ seconds), which is referred to as the observation period. Here, $T_{BW}$ is chosen to be 1 s.

Regarding the idle and busy states of wireless communication channels, IEEE 802.11 utilizes both physical-carrier sensing and virtual-carrier sensing for determination [46]. Physical-carrier sensing involves energy detection and carrier sensing, while the 802.11 standard employs the network allocation vector (NAV) to implement virtual-carrier sensing. In this study, the wireless channel is considered idle when both the receiving and transmitting states of a node are idle, and the elapsed time exceeds the value set by the NAV. Conversely, the wireless channel is considered busy when the node is in a receiving or transmitting state, or the elapsed time is less than the value set by the NAV.

The counting of the occupation factor COF of the channel is accomplished according to Equation (10):

(10) $$COF\left(v\right)=\frac{channel\_busy(T_{BW})}{T_{BW}}$$

**Figure 7:**
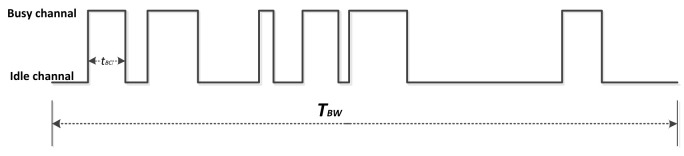
Busy channel.

According to Figure 7, channel_busy is determined by Equation (11):

(11) $$channel\_busy\left(T_{BW}\right)=\sum_{i}t_{BC_{i}}$$

To ensure an accurate assessment of the COF, a windowed mean method is employed for calculation. Hosts estimate their available bandwidth for new data transmissions as the product of channel bandwidth and the ratio of idle time to total time. Therefore, the available bandwidth factor can be conveniently defined as Equation (12):

(12) $$ABWF\left(v\right)=\frac{available\_bandwidth}{channel\_bandwidth}=1-COF\left(v\right).$$

### 4.5. Routing Neighbor Discovery

Vehicle nodes periodically broadcast beacons, and the receiving vehicle uses beacon information to update the link quality information between the sender and receiver, thereby establishing a neighbor table. The time interval for this process can be fine-tuned based on system requirements or network conditions. Algorithm 1 outlines the handling of vehicle beacon messages by the EARR protocol. In the third stage, neighbor messages are extracted and employed to update the neighbor node table. If the neighbor’s address is found within the received neighbor beacon, the node’s data is updated. Otherwise, a general neighbor entry is added, using the neighbor’s address as the index, and the link-stability factor, link-duration factor, received signal-strength factor, and available-bandwidth factor are calculated.
**Algorithm 1** Beacon exchange for neighbor discovery**Input:**  Graph G=(V,L), $P_{v_{n_{i}}}^{t_{i}}$, $v_{n_{i}}$, and Beacon **Output:**  Neighbor tables**Phase 1: Broadcast Beacon** 1:**for **$each\;v_{n_{i}}\in V,\;i=1,2,\cdots ,\left|V\right|$** do**2:   Broadcast(Beacon)3:**end for****Phase 2: Location estimation** 4:**for **$each\;v_{n_{i}}\in V,\;i=1,2,\cdots ,\left|V\right|$** do**5:   **if** vehicle $v_{n_{i}}$ position update time arrived **then**6:     $v_{n_{i}}$ obtains its location via GPS7:   **end if**8:**end for**  **Phase 3: Neighbor vehicle discovery** 9:**for **allreceivedBeacon** do**10:   Gets the source node address $ADDR(v_{n_{i}})$ contained in the beacon11:   **if** $ADDR\left(v_{n_{i}}\right)\in N_{ADDR}$ **then**12:     Update $v_{n_{i}}$ neighbor record13:   **else**14:     Add a new record for neighbor $v_{n_{i}}$15:   **end if**16:   Calculate link stability factor using Equation (5)17:   Calculate link duration factor using Equations (6)–(8)18:   Calculate received signal strengh factor using Equation (9)19:   Calculate available bandwidth factor using Equations (10) and (12)20:   Keep the $Q_{max}\left(v_{n_{i}}\right)$ value21:   Keep the beacon receiving time $BRT(v_{n_{i}})$22:**end for**

### 4.6. Adaptive Q-Learning Parameters

In Q-learning, the learning rate controls the step size at which the RL algorithm updates its value function or policy. It determines the extent to which newly acquired information supersedes old information. A higher learning rate leads to faster updates of Q values. The learning rate typically falls within the range of 0 to 1. Most existing Q-learning-based routing protocols have fixed learning rates. However, in VANETs, the links between vehicles can be highly unstable. Therefore, this study dynamically adjusts the learning rate with relative velocity adaptively. In this study, the adaptive learning rate (α) is controlled by a linear function of the stability of the link from node *v* to node *w* and is defined as follows:

(13) $$\alpha =\alpha_{min}+\left(\alpha_{max}-\alpha_{min}\right)\times LSF\left(l\right),$$

where $\alpha_{min}$ serves as the lower threshold for the learning rate, thereby ensuring a foundational update rate, while $\alpha_{max}$ functions as the upper threshold for the learning rate, thereby offering a degree of constraint.

The discount factor controls the extent to which a reinforcement-learning algorithm prioritizes future rewards. The range of values for the discount factor lies between 0 and 1. When γ = 0, it implies a focus solely on immediate rewards, while γ = 1 indicates the full consideration of future rewards. The discount factor plays a crucial role in balancing immediate rewards and future rewards.

The adjustment of the discount factor should be balanced according to the specific problem at hand. If future rewards significantly influence current decisions in the task, then the discount factor should be set to a higher value. Conversely, it should be reduced to a lower value if future rewards have less impact. In the context of VANET routing decisions, having reliable neighbors for data-packet forwarding represents a higher level of influence on current decision making. Therefore, it is necessary to increase the discount factor. The discount factor is defined as follows:

(14) $$\gamma =LDF\left(l\right)\times RSSF\left(l\right)\times ABWF\left(v\right)\times \gamma_{max},$$

where LDF is the link duration factor defined by Equation (8), RSSF is the received signal-strength factor defined by Equation (9), and ABWF is the available bandwidth factor defined by Equation (12). $\gamma_{max}$ indicates the upper limit constraint of discount factor. The value ranges from 0 to 1. It is evident from Equation (14) that to ensure stable data transmission, all three factors, namely LDF, RSSF, and ABWF, should be taken into account. A longer link duration factor ensures that communication remains within a reliable range, a larger received signal-strength factor minimizes environmental interference, and a higher available-bandwidth factor guarantees a better communication environment.

### 4.7. Reward Function and Penalty

The reward function is a critical parameter in the Q-learning algorithm, which is denoted as $r_{t}$. It signifies the reward that the agent obtains when transitioning from the current state $s_{t}$ to the next state $s_{t+1}$ after executing an action. The agent’s actions are guided by the reward function, and the goal of Q-learning-based routing algorithms is to maximize the reward by successfully transmitting data from the source to the destination. Therefore, the ability to accurately find the destination depends entirely on the reward function. The reward function is defined as follows:

(15) $$r_{t}=\left\{\begin{array}{cc} LDF(l)\times RSSF(l)\times \varphi , & \mathrm{if}\;s_{t+1}\;\mathrm{is}\;\mathrm{destination}\\ 0, & \mathrm{otherwise} \end{array}\right..$$

In order to ensure that the nodes are within the stable communication range of the destination node and have a sufficient signal strength, LDF and RSSF are introduced, with φ serving as a reward factor. If the next-hop node to which a data packet is forwarded is the destination node, the reward is LDF(l)×RSSF(l)×φ. Otherwise, the reward is 0. Q-learning is capable of establishing an effective path by providing rewards. To further enhance the effectiveness of finding the best forward relay node, a forward-oriented reward function has been introduced. The reward function can be redefined as follows:

(16) $$r_{t}=\left\{\begin{array}{cc} LDF(l)\times RSSF(l)\times \varphi , & \mathrm{if}\;s_{t+1}\;\mathrm{is}\;\mathrm{destination}\\ max\left(0,1-\frac{DIST^{t_{i}}\left(w,dest\right)}{DIST^{t_{i}}\left(v,dest\right)}\right)\times \phi ,\;w\in N_{v} & \mathrm{otherwise} \end{array}\right.,$$

where $DIST^{t_{i}}\left(w,dest\right)$ represents the distance from the next-hop node to the destination node. If the next-hop node’s position is closer to the destination node than the current position, the reward is calculated based on the forwarding progress. ϕ serves as a constraint factor, where ϕ≪φ, thereby restraining the forward-oriented reward function to avoid disrupting the reward effect of the target node.

The proposed protocol updates the Q table through broadcast, thereby preventing the occurrence of routing voids, as there is no requirement for an exploration and exploitation process. This is due to the difficulty of adapting routing exploration and exploitation processes to rapid changes in the network topology, which can easily lead to local optima or link disconnections. Periodic beacons allow for adaptive responses to environmental changes, thereby automatically adjusting the route selection. However, even in this update process, a specific error scenario can still arise, where nodes consider each other as the next-hop node with the highest Q value. To prevent this error, we introduce the penalty value −ψ, where ψ>0:

### 4.8. Q-Table Update

Algorithm 2 delineates the process of updating the Q table for vehicles. In Algorithm 1, critical factors such as link stability, link duration, received signal strength, and effective bandwidth are computed using beacon information. Algorithm 2 employs this information to calculate α, γ, and $r_{t}$. And using the way of broadcasting, we calculate the Q value of each neighbor. Beacon messages are broadcast at fixed intervals, without the use of adaptive interval timing. This choice is made because adapting interval times in the VANET environment requires frequent adjustments, which can easily lead to a deterioration in communication conditions in certain areas and increased channel congestion. The degradation in communication quality caused by control message overhead is not reasonable. Moreover, determining the appropriate adaptive interval is a challenging problem. A fixed beacon interval cannot accommodate the varying degrees of changes in parameters such as speed, signal strength, and bandwidth. Therefore, the extent of each Q-learning parameter update must be given careful consideration.

The computation of α and γ is crucial for determining Q values, and each Q-value update represents an adaptation to the environment. For high-speed mobile nodes, the link duration adjusts based on relative motion, thereby periodically adapting to the movement of neighboring nodes according to the beacon’s intervals. Regarding the perceived signal strength, further adjustments are made to select neighboring nodes. When the signal strength is not suitable for the next-hop selection, the future reward is reduced. Conversely, in a well-performing communication environment, the future reward is maximized. The same considerations can be applied to bandwidth. The dynamic discount factor γ ensures that the data packets transmitted over the path have communication quality as close as possible to the optimal. Properly adjusting these variable parameters impacts the quality of data transmission.
**Algorithm 2** Updating Q-table values for each vehicle**Input:** Neighbor tables **Output:**  Q-tables1:Initialize the Q-tables for each vehicle2:**for **$each\;v_{n_{i}}\in V,\;i=1,2,\cdots ,\left|V\right|$** do**3:   **for** $each\;v_{n_{j}}\in N_{v_{n_{i}}},\;j=1,2,\cdots ,\left|N_{v_{n_{i}}}\right|$ **do**4:     **if** $\left|Current\_time-BRT\left(v_{n_{j}}\right)\right|\geq \tau_{B}$ **then**5:        Delete neighbor record6:     **else**7:        Calculate α using Equation (12)8:        Calculate γ using Equation (13)9:        Calculate reward $r_{t}$10:       Extracted the $Q_{max}\left(v_{n_{j}}\right)$ from neighbor $v_{n_{j}}$11:       **if** $v_{n_{j}}\;was\;recorded\;for\;the\;first\;time$ **then**12:          $Q(v_{n_{i}},v_{n_{j}})=0$13:       **else**14:          $Q\left(v_{n_{i}},v_{n_{j}}\right)=Q_{old}\left(v_{n_{i}},v_{n_{j}}\right)$, Records from the Q-tables15:        **end if**16:        Update q values as follows:17:        $Q\left(v_{n_{i}},v_{n_{j}}\right)=Q\left(v_{n_{i}},v_{n_{j}}\right)+\alpha \times \left(r_{t}+\gamma \times Q_{max}\left(v_{n_{j}}\right)-Q\left(v_{n_{i}},v_{n_{j}}\right)\right)$18:     **end if**19:   **end for**20:**end for**  **Calculate reward**21:**if **$v_{n_{j}}\;is\;recorded\;as\;BS$** then**22:   Calculate $r_{t}$ using Equation (14)23:**else if** nodesbothconsidereachotherasthenext−hopnodewiththehighestQ−value**then**24:   $r_{t}=-\psi$25:**else**26:   Calculate $r_{t}$ using Equation (15)27:**end if**

### 4.9. EARR Routing Selection

In the proposed enhanced adaptive Q-learning-based routing (EARR) approach, the vehicle nodes’ position can be obtained by GPS. EARR utilizes Algorithm 1 to manage a neighbor table that contains status information about neighboring nodes. This table is regularly updated through the exchange of beacon messages to adapt to the highly dynamic network environment. EARR also divides routing paths based on the vehicle environment. Finally, Q-learning is used to make routing decisions. Additionally, Algorithm 2 is employed to construct the EARR neighbor Q table, which takes into account parameters such as link stability based on the learning rate, link duration based on the discount factor, signal strength, and effective bandwidth. Furthermore, a forward-oriented reward is incorporated into the reward function.

The source vehicle generates data packets and determines the destination’s location. If the destination node (dest) is within the range of vehicle *v*, the Q(v,dest) is greater than the Q value of any other neighboring node, and the record of the destination node is not expired, then the data packet is transmitted to the destination node. Otherwise, the source vehicle will select and forward the data packet among its neighboring nodes. To limit data-packet transmission, a time-to-live (TTL) mechanism is introduced. If TTL equals 0, the data packet is discarded. If the neighbor table is empty, the data packet is also discarded. If the neighbor table is not empty, the node uses Q-learning to forward the data packet. To ensure that data packets are not transmitted redundantly, EARR recommends maintaining a path recording mechanism. If the neighbor node with the highest Q value has already been recorded, then the second-highest Q value neighbor is chosen. If that neighbor is also recorded, the process continues iteratively. This is shown in Algorithm 3.
**Algorithm 3** Routing-path selection model for each vehicle**Input:** Q-tables **Output:**  The next hop neighbor vehicle node is selected1:**while** DatapacketsDPthatneedtobetransmitted **do**2:   **if**  Disinitsowntransmissionrange and$Q\left(v,dest\right)\geq \max_{j}(Q\left(v,v_{n_{j}}\right),\;j=1,2,\cdots ,\left|N_{v}\right|\;and\;\left|Current\_time-BRT\left(v_{n_{j}}\right)\right|\;<\;\tau_{B}$ **then**3:     Transmit data packets DP to dest4:   **else**5:     **if** TTL(DP)−1=0 **then**6:        Delete data packets DP
7:     **else if** $|N_{v}|=0$ **then**8:        Delete data packets DP9:     **else**10:        **for** $j=1,2,\cdots ,|N_{v}|$ **do**11:          Obtain the address of the next hop neighbor node with Q size *j*-th12:          **if** $v_{j-th}\in \mathrm{RP}\left(src,v\right)$ **then**13:             Continue14:          **else**15:             Record $v_{j-th}$ to RP(src,v)16:             Transmit data packets to $v_{j-th}$17:          **end if**18:        **end for**19:        **if** $all\;v_{n_{j}}\in \mathrm{RP}\left(src,v\right),\;j=1,2,\cdots ,\left|N_{v}\right|$ **then**20:          Delete data packets DP21:        **end if**22:     **end if**23:   **end if**24:**end while**

## 5. Simulation Results and Analysis

### 5.1. Simulation Environment and Parameters

This study conducted extensive simulations using the SUMO, Veins, and OMNeT++ toolset. OMNeT++ is an object-oriented discrete event network simulator that focuses on simulating wireless network communication. It allows us to model and analyze various communication protocols and network topologies, thereby making it particularly suitable for researching wireless communication systems. SUMO is a traffic simulator that encompasses vehicle driving patterns, behaviors, and route planning. It enables us to generate realistic traffic scenarios that can be used in conjunction with other external programs. Veins is an open-source framework for simulating vehicular networks. It is built on top of OMNeT++ and SUMO. The simulator instantiates an OMNeT++ node for each vehicle in the simulation and pairs the node’s movement with vehicle movements in the road-traffic simulator (i.e., SUMO). Veins provides a well-developed underlying structure for vehicular wireless networks, including the physical layer and MAC layer based on the 802.11p protocol. This makes it convenient for secondary development, such as improving MAC layer protocols or researching routing protocols.

The simulation scenario covers a 2 km × 2 km urban area, consisting of 40 bidirectional two-lane road segments and 25 intersections.

In the algorithm, vehicles adapt to their environment using Q-learning to obtain the routing Q table. In our approach, the lower limit of the learning rate for Q-learning was set to 0.6, and the upper limit was 0.95. The upper limit for the discount factor was 0.7. These values needed to be adjusted based on the operational environment of the vehicles. The parameters used in the simulations are listed in Table 2. To accommodate the characteristics of vehicle mobility, this study did not impose distance restrictions between vehicles. Vehicle flow was generated as random traffic by SUMO, with vehicle speeds ranging from 0 to 50 km/h, which aligns with the typical characteristics of urban traffic.

**Figure 8:**
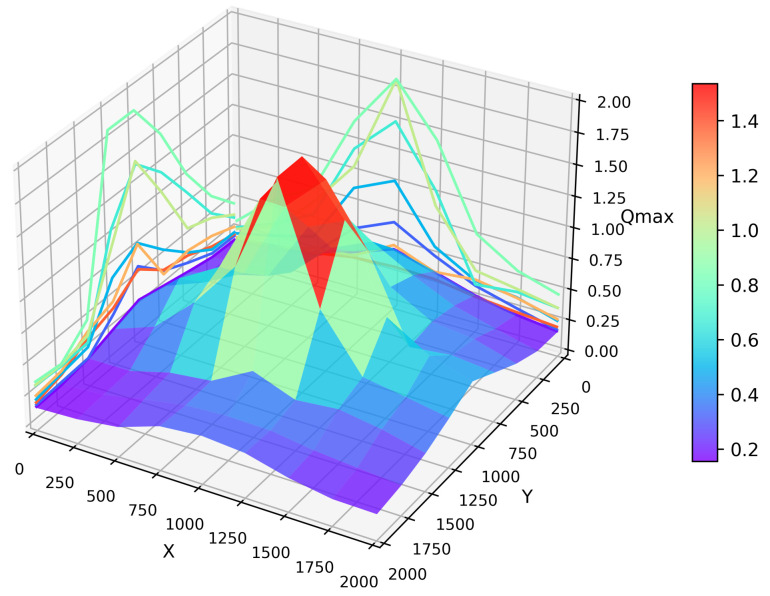
$Q_{max}$ convergence terrain map.

In the simulation, to observe the convergence capability of each region, the relationship between the location and $Q_{max}$ is presented in Figure 8. At a randomly chosen moment, the $Q_{max}$ values were retained for all the vehicles in the entire simulated map. The average $Q_{max}$ for all the vehicles within a fixed radius was computed as the $Q_{max}$ for that region. From the graph, we can observe that as the training process of the Q table for the entire network progressed, the closer a region was to the destination area, the larger the average $Q_{max}$ for that region, thereby ensuring the formation of links. The projections in the x and y directions also clearly show the variations in $Q_{max}$.

An interesting phenomenon can be observed in the graph along the periphery of the entire area; when vehicles maintained a straight-line road connection with the destination region, their $Q_{max}$ value was maximized at these edge points, such as at coordinates (2000, 1000) in the graph. This is understandable because maintaining a straight-line road to the destination region minimizes the number of hops in routing, thereby resulting in minimal convergence loss. In contrast, in other areas with urban road environments, the number of hops in routing is bound to increase.

**Figure 9:**
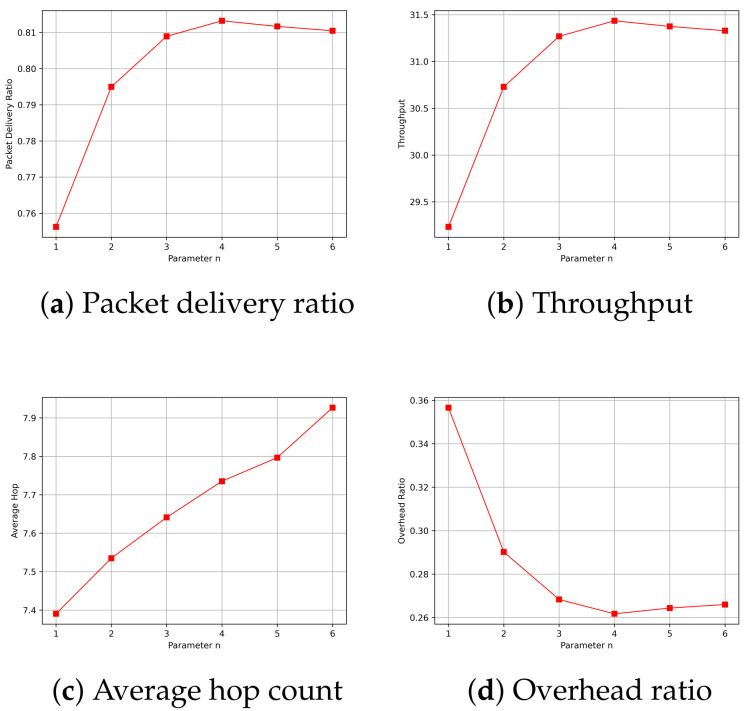
Performance of different parameters of *n*.

In the experiments, it was necessary to determine the variable parameters in the routing algorithm based on the simulation environment. When calculating the received signal-strength factor, $RSSI_{max}$ and $RSSI_{min}$ needed to be determined. $RSSI_{max}$ was determined based on the average statistically calculated RSSI under interference-free conditions at the limit communication distance. $RSSI_{min}$, on the other hand, needed to be adjusted based on the simulation and was determined empirically. When calculating the link-duration factor, the parameter *n* needed to be determined. This parameter was determined by simulating the packet delivery rate, throughput, average hop count, and overhead ratio. From Figure 9, it can be observed that when *n* equaled 4, 5, and 6, the packet delivery rate, throughput, and overhead ratio all achieved satisfactory optimal values. However, with the increase in *n*, the average hop count also gradually increases. Therefore, in this context, n=4 represents the most ideal choice. Consequently, this result was used for all subsequent simulations.

This study compared the dynamic and static learning rate of the EARR with the GPSR [13] and CAEB [40], and itanalyzed the simulation results. The following metrics were used to evaluate the performance of the routing protocols:

- Packet Delivery Ratio: The ratio of the total number of packets received at the destination node to the total number of packets generated by the source node.
- Throughput: Throughput refers to the amount of data or the number of data packets successfully transmitted within a unit of time.
- Average Hop Count: The average number of nodes that a packet passes through on the routing path from the source node to destination node.
- Standardization Delay: The average end-to-end time refers to the time it takes for data to be transmitted from the source node to the destination node along the routing path. To fairly assess the delay experienced by mobile nodes, delay is standardized based on the length of the routing path.
- Overhead Ratio: The total number of generated packets minus the numbers of packets successfully received by the destination and then divided by the numbers of packets successfully received by the destination.
- CBR Flows and Constant Bit Rate (CBR) traffic: During the simulation process, the source nodes for each CBR flow are initially randomly selected by the program and remain unchanged during the simulation. In this context, CBR flow simulation involves an increase from 10 to 60 nodes, with each node sending four data packets of 512 bytes per second.

Each data point in the graphs is the average of simulation runs with different random seeds. Each simulation lasted for 400 s. In each simulation, the source node needed a warm-up time before starting to transmit packets and ended transmitting packets 20 s before the end of the simulation.

### 5.2. Simulation Results and Analysis

#### 5.2.1. Impact of Simulation Time

**Figure 10:**
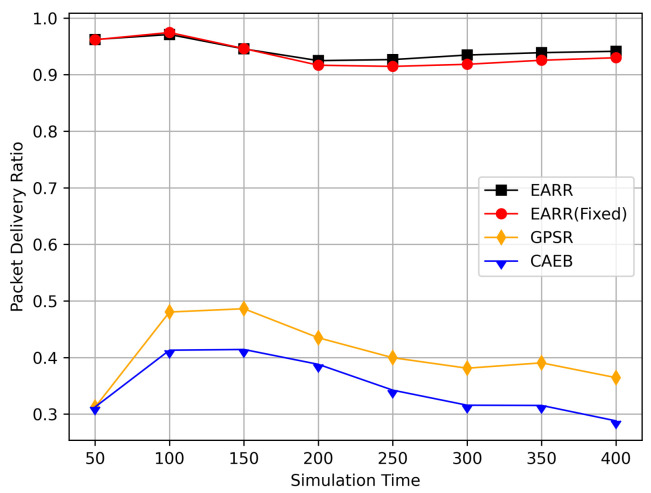
Packet delivery ratio versus simulation time.

**Figure 11:**
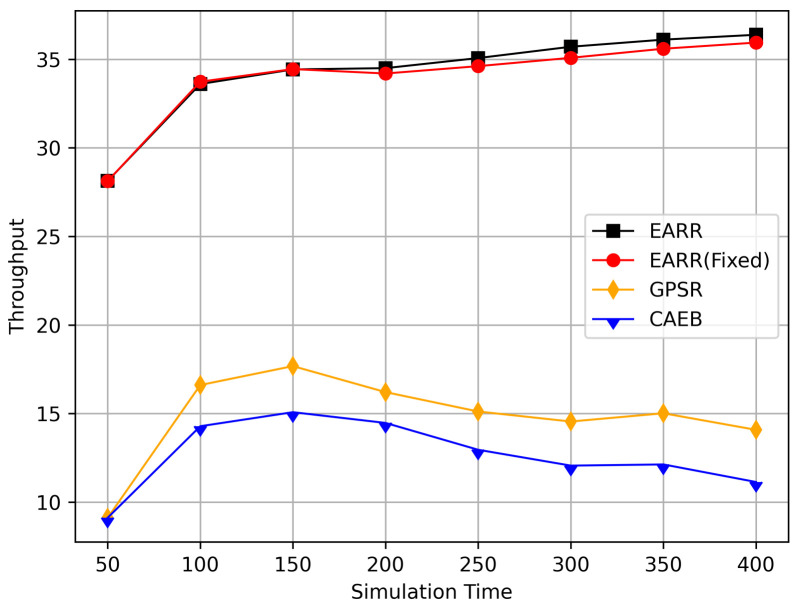
Throughput versus simulation time.

**Figure 12:**
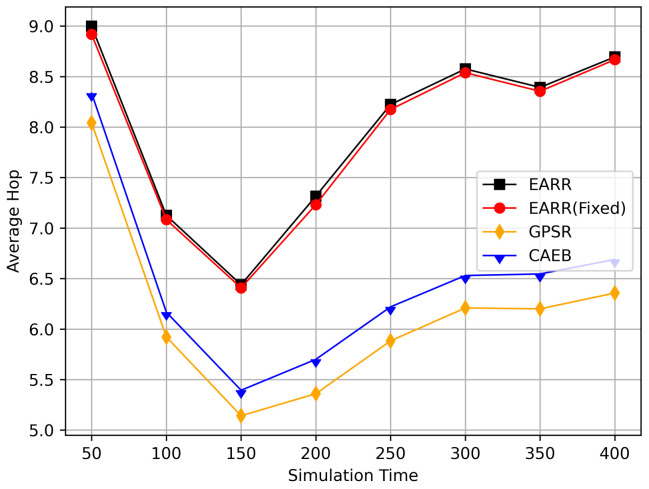
Average hop count versus simulation time.

**Figure 13:**
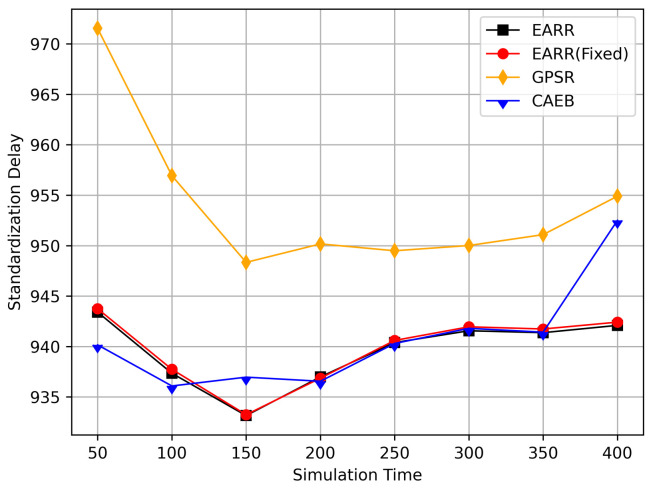
Standardization delay versus simulation time.

**Figure 14:**
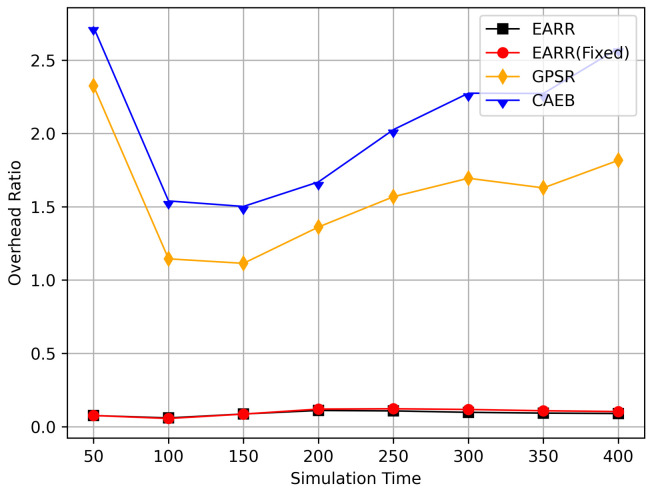
Overhead ratio versus simulation time.

In this simulation, vehicles moved rapidly on urban streets while adhering to traffic regulations. Source nodes attempted to send data packets at fixed intervals, and the performance was observed at different time points. Figure 10, Figure 11, Figure 12, Figure 13 and Figure 14 illustrate various performance metrics, including packet delivery rate, throughput, hop count, standardized latency, and overhead ratio. At different time points depicted in the graph, vehicles moved to different positions. When the time point reached 50, the Euclidean distance from the destination was 855 m. The time points 100, 150, 200, 250, 300, 350, and 400 correspond to 515, 706, 1158, 1288, 1015, 1081, and 1400 m, respectively.

At different moments, source nodes moved to different areas, thereby representing the need for routing protocols to adapt to changing environments. Figure 10 illustrates the relationship between the packet delivery rate and simulation time. EARR comprehensively considers various environmental parameters to select the optimal next hop. Meanwhile, GPSR implements a greedy strategy throughout the entire area, where intersections and buildings may lead to signal attenuation and packet loss. CAEB employs fuzzy logic to select edge nodes, and data packets are forwarded via these edge nodes. The proper selection of edge nodes has a significant impact on the protocol’s performance.

As simulation time goes on, vehicles travel along the streets. Compared to the GPSR and CAEB routing protocols, our proposed EARR protocol demonstrated significant performance improvement in the packet delivery ratio (PDR). Environmental changes were evident from the GPSR’s PDR, where at the start of the simulation, source nodes gradually moved closer to the destination and then moved away again. Undoubtedly, the PDR significantly improved when the nodes were closer. The graph illustrates that EARR can adapt to the environment throughout the entire simulation process, thereby maintaining a consistently high PDR. It does not experience performance fluctuations due to environmental changes, thereby showing excellent robustness. The GPSR, relying solely on node location information without considering other environmental factors, experienced decreased performance. The CAEB struggled with selecting edge nodes that could adapt to highly dynamic vehicular flows, and it also did not take other environmental factors into account.

Figure 11 illustrates the relationship between the throughput and simulation time. Throughout the entire simulation timespan, EARR consistently outperformed the other protocols in terms of throughput. The throughout remained stable and showed a slight trend of increasing over time, as illustrated by the changes of the nodes’ positions and the other environmental conditions. In contrast, the GPSR and CAEB were significantly impacted by environmental factors. Their throughputs improved only when the nodes were closer to the destination. Once the distance increased, the lower PDR substantially hampered the throughput performance. Our protocol took into account the relative movement between nodes. Therefore, regardless of how vehicles moved, each routing decision ensured that vehicles remained within a reliable communication distance.

Figure 12 displays the average hop count at different simulation time points. The EARR algorithm exhibited an average hop count that was one to two hops higher than the other routing protocols. This occurrence is unavoidable, because the EARR protocol must select the next-best hop to forward the data packets, thereby leading to the abandonment of many unstable nodes. In contrast, the GPSR always chose the node closest to the destination without considering the node’s communication environment. The EARR protocol selected edge nodes within the R/2 radius, ensuring a one-hop distance. As the time progressed, the gap in the hop counts became significantly pronounced in the graph. This is because the EARR protocol guarantees a consistently high packet arrival rate and throughput, thereby resulting in busy communication on the routing paths. Additionally, the protocol considers the network’s available bandwidth, which means some nodes might not be chosen for a period due to busy communication. This limitation restricts the characteristic of EARR forming the shortest path.

Figure 13 displays the standardized delays at different simulation time points. It can be observed that there is little difference in delays among the various protocols; however, our protocol consistently maintained the lowest standardized delays. The standardized delays of each protocol varied with changes in the vehicle environment during different simulation times. As the nodes gradually approached, the delays decreased, but as the distance increased, the delays also increased. Utilizing standardized delays allows for a better evaluation of latency performance under different environmental changes. In Figure 11, it is evident that numerous packets were accepted when they were closer to the destination, thereby leading to higher throughput. However, once the distance increased, the throughput decreased. Therefore, packets closer to the destination contributed significantly to the calculated performance metrics, and our protocol consistently ensured high throughput. This is why we standardize packet delays based on the routing length.

The EARR protocol demonstrated the highest packet delivery rate and required only one second beacon intervals to establish routes. Therefore, EARR stands out as the most efficient routing protocol, thereby boasting low communication overhead. The relationship between the overhead ratio and simulation time is depicted in Figure 14. The overhead ratio varied with the movement of vehicles: the closer the distance, the faster the route establishment, thereby resulting in a lower overhead ratio. The EARR protocol ensures rapid route establishment in any environment, coupled with a higher message delivery rate, which minimizes the number of discarded messages, thereby consequently reducing the overhead ratio. Messages that are discarded consume network resources during transmission but do not achieve the intended goal of delivering useful information, thereby increasing communication overhead.

#### 5.2.2. Impact of Data Interval

In this simulation, the source node attempted to send packets at different rates to further observe the routing performance.

**Figure 15:**
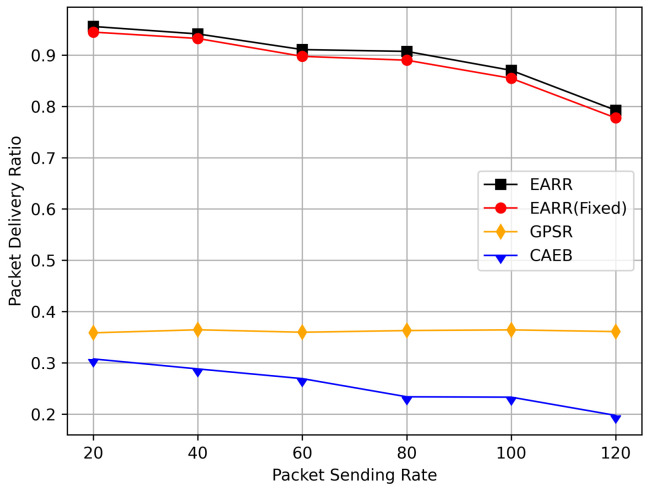
Packet delivery ratio versus packet sending rate.

Figure 15 illustrates the packet delivery rates at different transmission rates. As the message transmission rate increased, the number of generated packets within a certain period also rose. With the passage of simulation time, the increasing number of packets burdened the network. EARR performed exceptionally well at various transmission rates; the protocol evaluated the effective bandwidth and could adapt to network environment changes in real time.

**Figure 16:**
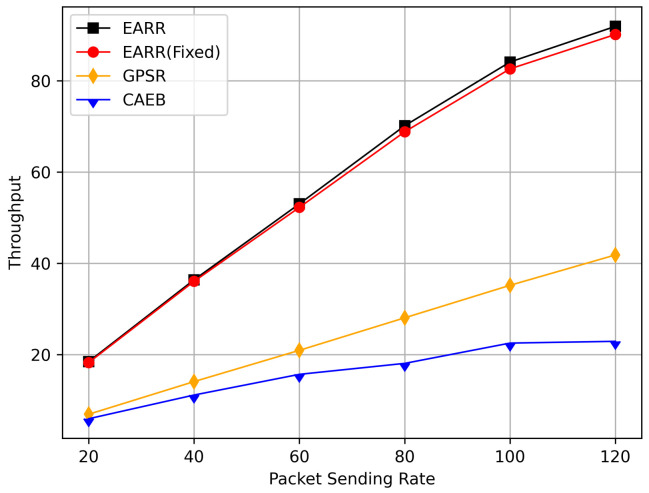
Throughput versus packet sending rate.

Figure 16 demonstrates the throughput at different transmission rates. As the transmission rate increased, the throughput also grew correspondingly. The EARR maintained a consistent linear growth trend at various transmission rates, thereby indicating its ability to adapt to different network congestion levels. The throughput of the EARR protocol was more than double that of the GPSR and CAEB. Vehicles could adapt to varying mobility, interference, and network states. Due to the low packet loss rate in the EARR, the EARR showed significant performance improvements in throughput.

**Figure 17:**
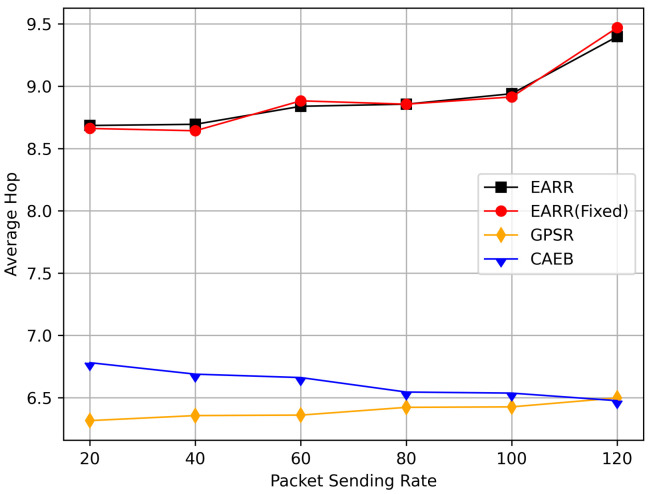
Average hop count versus packet sending rate.

**Figure 18:**
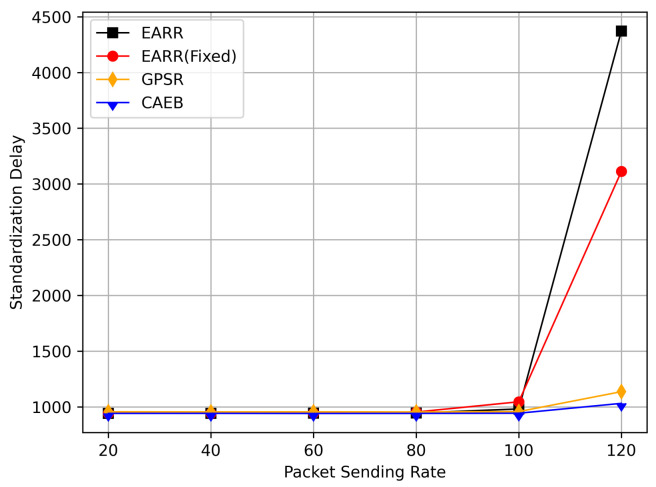
Standardization delay versus packet sending rate.

Figure 17 demonstrates the average hop count at different transmission rates. Similarly, to ensure high PDR and throughput, the routing hop count increases. Therefore, the average hop count in EARR is higher than that in other protocols. Analyzing the routing performance over simulation time reveals that high PDR and throughput are maintained only when the destination is nearby. Consequently, GPSR consistently maintains a very low hop count throughout the simulation, while the proposed protocol experiences a higher average hop count, leading to increased latency due to the additional hops. Considering results from Figure 18, the normalized delay of EARR is generally similar to that of GPSR and CAEB. Only when the transmission rate reaches 120, significant fluctuations occur in EARR. This phenomenon is a result of ensuring high PDR in high-load communication scenarios.

**Figure 19:**
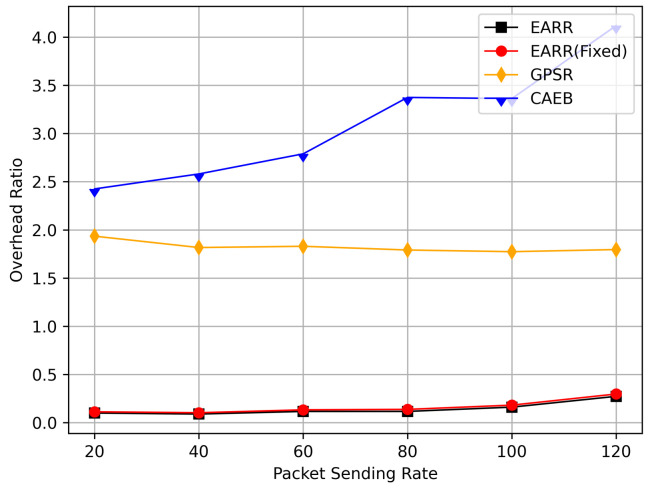
Overhead ratio versus packet sending rate.

Figure 19 illustrates the overhead ratio at different transmission rates. The EARR protocol demonstrated remarkable adaptability to varying network traffic loads, thereby consistently maintaining an extremely low overhead ratio. In contrast, the GPSR exhibited stable overhead ratios due to its higher packet-loss rate, thereby making it challenging for the network to experience high-load situations. The CAEB relied on edge nodes to forward packets, thereby keeping these nodes consistently busy and resulting in an increasing overhead ratio over time.

#### 5.2.3. CBR Flows

**Figure 20:**
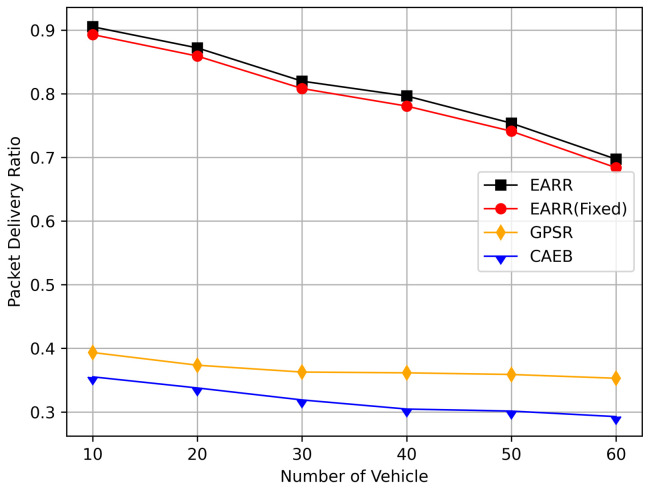
Packet delivery ratio versus CBR flows.

In this simulation, the performance of the routing under different CBR flows was analyzed. Figure 20 illustrates the packet delivery ratio (PDR) under different traffic loads. As the network congestion intensified with an increase in the number of flows, the PDR gradually decreased. However, the EARR consistently exhibited the highest PDR. With a flow count of 60, the EARR achieved a PDR of 69.7%, while the EARR (Fixed) maintained a PDR of 68.3%. In contrast, the GPSR and CAEB had PDRs of 35.3% and 29.2%, respectively. Throughout the simulation, the EARR maintained a high PDR in all regions, thus being unaffected by node mobility or environmental changes.

**Figure 21:**
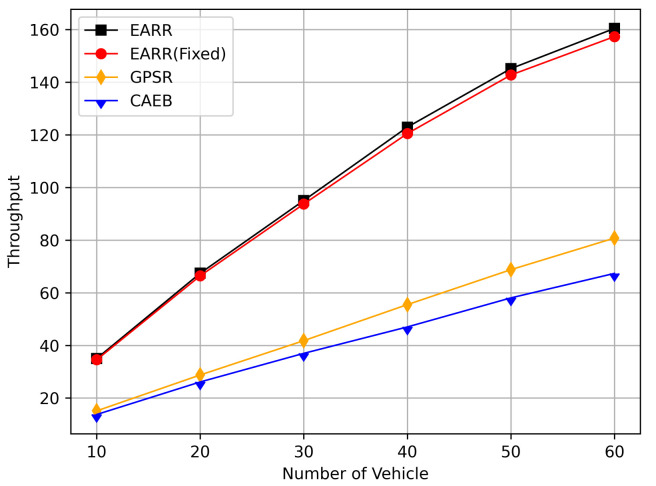
Throughput versus CBR flows.

As shown in Figure 21, the throughput increased with the rise in the number of flows. With more sending nodes, there was a rapid growth in the number of transmitted packets. Consequently, the network became more prone to congestion. The EARR is a location-based environment-aware routing protocol that utilizes the Q-learning approach to optimize bandwidth selection. By introducing the effective bandwidth factor into the discount factor, the EARR minimizes the overall route bandwidth. Since Q values are updated using broadcasting, the problem of the GPSR’s blind path problem is eliminated. In urban topology networks, addressing the blind path problem is challenging through perimeter forwarding. In congested network environments, the EARR exhibited a linear growth trend in throughput, thereby surpassing the other protocols significantly.

**Figure 22:**
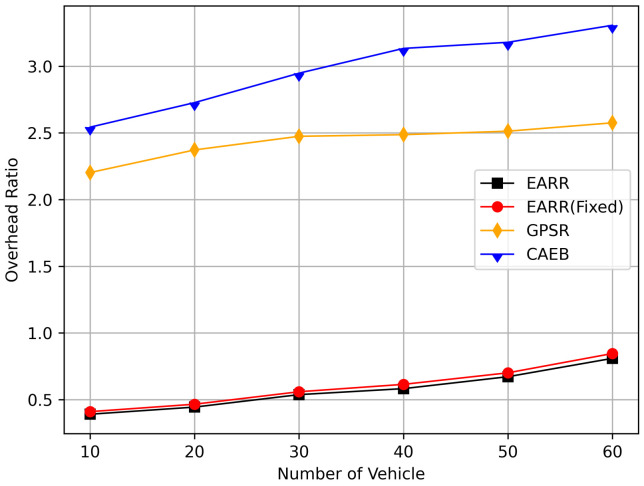
Overhead ratio versus CBR flows.

Figure 22 illustrates the relationship between the overhead ratio and the number of flows. The overhead ratio increased as the number of packets in the network grew. This is primarily because the more packets there are to be transmitted in the network, the greater the number of failed packet transmissions caused by network congestion. In comparison, the EARR consistently maintained the lowest overhead ratio and exhibited a minimal growth rate even as the number of flows varied.

## 6. Conclusions

This study proposed an environment-aware adaptive reinforcement routing (EARR) protocol. The EARR protocol effectively utilizes the unique characteristics of vehicular environments, such as dynamic changes in traffic patterns, urban environmental interferences, and fluctuations in communication channels, to make routing and forwarding decisions. This study employed a Q table instead of a routing table, thereby significantly reducing routing overhead, as there is no need for route discovery and maintenance. To enhance the efficiency and reliability of next-hop node selection, the EARR protocol utilizes geographical location change information of neighbors, thereby extending the local view of the network topology using Q-learning. This helps in choosing the optimal forwarding node to establish an optimal or suboptimal route. The neighbor table established through the broadcast mechanism requires the calculation of Q values for each entry using a discount factor determined based on link duration, received signal strength, and effective bandwidth. Adaptive Q-learning enhances routing decisions between the source and destination; the proposed Q-learning technique can adaptively adjust Q values based on changes in the network topology. Therefore, the proposed EARR protocol is more realistic when applied to VANETs. Simulation results demonstrate that our protocol performs well in terms of the PDR, throughput, normalized delay, and overhead ratio, thereby making it an efficient protocol.

As wireless technology improves by leaps and bounds, spectrum resources are becoming increasingly valuable. Therefore, in the future, the authors will not only consider making routing protocols adapt to more realistic working environments and more complex signal propagation models, but also place emphasis on optimizing resource management and scheduling in highly dynamic VANET environments.

## Figures and Tables

**Table 1:** Table of abbreviations and notations.

Notation	Description
MANET	Mobile ad hoc network
VANET	Vehicular ad hoc network
RSSI	Received signal-strength indication
OBU	On-board unit
V	Set of vehicle nodes
L	Set of links
RP(src, dest)	Routing path from the *S* to the *D*
#hops(RP)	Number of hops of the route RP
*v*, *w*	Vehicle node in the network
$v_{n_{i}}$	Vehicle with serial number $n_{i}$
$N_{v}$	Neighbor set of vehicle node *v*
$P_{v}^{t_{i}}$, $P_{w}^{t_{i}}$	The position of vehicle node *v*, *w* at time $t_{i}$: $P_{v}^{t_{i}}$ = ($x_{v}^{t_{i}}$, $y_{v}^{t_{i}}$), $P_{w}^{t_{i}}$ = ($x_{w}^{t_{i}}$, $y_{w}^{t_{i}}$)
DIST(·)	Euclidean distance
LSF(·)	Link stability factor
LD(·), LDF(·)	Link duration and link duration factor
RSSF(·)	Received signal-strength factor
COF(·)	Channal occupation factor
ABWF(·)	Available bandwidth factor
RV(·)	Relative velocity
BRT(·)	Beacon reception time
$\tau_{B}$	Neighbor information expiration interval
$T_{B}$	Beacon interval
ADDR(*v*)	The address of vehicle *v*
α, γ	Learning rate and discount factor

**Table 2:** Parameters used in simulations.

Parameter	Value
Simulation area	2 km × 2 km
Number of intersections	25
Number of segments	40
Number of vehicles	400
Signal transmission range	250 m
Packet size	512 bytes
Packet sending rate	20 ∼ 120 packets/sec
MAC protocol	IEEE 802.11p
Bit rate	6 Mbps
Path loss exponent	2
Propagation model	Free-space
Beacon interval	1 s
Simulation duration	400 s
$T_{BW}$	1 s
$\alpha_{max},\alpha_{min}$	0.95, 0.6
$\gamma_{max}$	0.7

## Data Availability

Data sharing not applicable.
